# Relationship Between Carotid Intraplaque Neovascularization and Immune–Inflammatory Biomarkers with Coronary Stenosis

**DOI:** 10.31083/RCM28171

**Published:** 2025-05-23

**Authors:** Yixue Wang, Jinhong Chen, Xuemin Li, Xinyu Tang, Yu Zhang, Xiao Yang

**Affiliations:** ^1^Department of Ultrasound, The Second People’s Hospital of Hefei, Hefei Hospital Affiliated to Anhui Medical University, 230011 Hefei, Anhui, China; ^2^The Fifth Clinical College of Medicine, Anhui Medical University, 230032 Hefei, Anhui, China

**Keywords:** carotid vulnerable plaque, Angio PLanewave UltraSensitive imaging, intraplaque neovascularization, immune inflammation, biomarker, coronary stenosis

## Abstract

**Background:**

Intraplaque neovascularization (IPN) correlates significantly with plaque vulnerability and can be detected using Angio PLanewave UltraSensitive imaging technology (Angio PL.U.S.; AP). Several immune–inflammatory biomarkers that reflect the state of inflammation and immune homeostasis in the body are currently used to assess cardiovascular and cerebrovascular diseases. This study aimed to investigate the correlation between carotid IPN scores and several immune–inflammatory indicators in patients with different degrees of coronary artery stenosis.

**Methods:**

This study prospectively enrolled 107 patients with coronary artery stenosis confirmed by coronary angiography (CAG). Preoperative ultrasonography was performed to screen for carotid plaques, and AP was conducted to determine whether IPN was present and correctly scored. The levels of immune–inflammatory indicators, plaques, and coronary artery lesions between groups with and without IPN and different IPN scores were analyzed. We utilized logistic regression models to determine the independent predictors of IPN and constructed receiver operating characteristic (ROC) curves. Odds ratios (ORs) and 95% confidence intervals (CIs) were calculated.

**Results:**

Differences in systemic immune inflammation index (SII) levels and plaque thicknesses were found between the groups with and without IPN and between different IPN scores (*p* < 0.05). The IPN scores were positively correlated with SII levels (r = 0.268, *p* = 0.005), plaque thickness (r = 0.273, *p* = 0.005), and Gensini score (r = 0.446, *p* < 0.001). SII levels (per 10-unit increase) (OR = 1.031) and plaque thickness (OR = 1.897) were independent risk factors for IPN. When the SII was 541 × 10^9^/L and the thickness of the plaque was 2.25 mm, the area under the curve (AUC) was 0.653 and 0.656, respectively. The AUC of the combined diagnosis was 0.711.

**Conclusion:**

Elevated SII levels and increased plaque thickness were associated with the vulnerability of carotid plaques in patients with coronary artery stenosis and may signal increased coronary artery stenosis.

**The Clinical Trial Registration:**

ChiCTR2400094458, https://www.chictr.org.cn/hvshowprojectEN.html?id=266292&v=1.0.

## 1. Introduction

Coronary artery disease (CAD) is among the most prevalent cardiovascular conditions, accounting for a significant number of deaths globally [1]. Atherosclerosis (AS) characterized by systemic chronic inflammation within the vasculature, frequently manifests as coronary atherosclerosis. This condition leads to luminal narrowing or blockage in the coronary arteries, resulting in myocardial ischemia and infarction [2]. With disease progression, fibrous plaques form on arterial walls, which are prone to calcification, ulceration, thrombosis, and intraplaque hemorrhage. Plaque rupture can precipitate severe cardiovascular events, including myocardial infarction and stroke [3]. The carotid artery serves as a vital indicator of systemic arterial health, and its assessment is crucial for gauging the overall vascular health. The stability of carotid plaques, therefore, indirectly predicts the risk of cardiovascular and cerebrovascular diseases.

Previous studies have shown that intraplaque neovascularization is significantly correlated with plaque vulnerability and is considered the most powerful independent predictor of plaque rupture and bleeding [4]. In addition, previous studies have also found that a higher level of carotid intraplaque neovascularization (IPN) assessed by contrast-enhanced ultrasound (CEUS) is related to coronary artery disease (stenosis ≥50%), providing a new perspective for the assessment of coronary artery disease [5]. CEUS is an invasive examination. Although it can provide more accurate information on dynamic blood flow, its operation is relatively complex, requires the injection of contrast agents, has the risk of allergic reactions, and is more costly [6]. These factors have limited its wide application in clinical medicine. Angio PLanewave UltraSensitive imaging (Angio PL.U.S.; AP) is a high-resolution non-invasive examination that can quantitatively estimate the blood flow within the blood vessels [7]. A histological study has confirmed that IPN detected by CEUS is closely related to the number of newly formed microvessels in the adventitial vasa vasorum of histological specimens [8]. In addition, a comparative study has shown that AP technology and CEUS have been consistent in assessing IPN and further verify the effectiveness of AP technology in assessing the formation of IPN [6].

AS is a chronic immune inflammatory process involving a variety of immune cells and inflammatory mediators, which collectively contribute to the instability of plaque structure. Although previous studies have highlighted the association between IPN and plaque vulnerability, research on the correlation between potential serological markers of IPN and plaque vulnerability with the degree of coronary artery stenosis is still relatively scarce [4]. Given the simplicity and accessibility of peripheral blood immune cell testing, this study aims to explore the correlation between IPN detected using AP technology and several immune inflammatory indicators in patients with varying degrees of coronary artery stenosis. This research could provide valuable insights for the early identification and intervention in coronary artery disease.

## 2. Materials and Methods

### 2.1 Study Design and Population

This prospective study consecutively enrolled 107 patients who underwent coronary angiography (CAG) at the Department of Cardiology at the Second People’s Hospital of Hefei between December 2023 and May 2024. There were 64 males and 43 females, with ages ranging from 37 to 88 years old. The inclusion criteria were: (1) age ≥18 years; (2) clinical indication for coronary angiography to assess CAD, with confirmed coronary artery lesions by CAG; (3) no clinical contraindications for CAG; (4) local carotid intima-media thickness (IMT) ≥1.5 mm. The exclusion criteria were: (1) history of carotid endarterectomy or percutaneous coronary intervention or coronary artery bypass grafting; (2) presence of diseases associated with thickening of the carotid IMT (such as immune diseases, arteritis); (3) presence of other cardiac diseases (such as valvular heart disease, myocarditis, dilated cardiomyopathy, hypertrophic cardiomyopathy, known or suspected cardiac shunts); (4) severe hepatic or renal insufficiency, systemic infectious diseases, or coagulation disorders; (5) incomplete clinical data; (6) poor quality of carotid ultrasound images.

### 2.2 Clinical Data Collection

Clinical baseline data was collected within 24 h of patient admission, including demographic characteristics: sex, age, Body Mass Index (BMI), atrial fibrillation, stroke, hypertension, hyperlipidemia, diabetes, smoking history, drinking history, history of statin use; laboratory indicators: peripheral venous blood was collected from all patients, including neutrophils (N), lymphocytes (L), monocytes (M), and platelets (P), as well as levels of fasting blood glucose (FBG), triglycerides (TG), total cholesterol (TC), low-density lipoprotein cholesterol (LDL-C), high-density lipoprotein cholesterol (HDL-C), uric acid (UA), and creatinine (Cr); imaging indicators: IPN score, IMT, plaque length, plaque thickness, plaque echogenicity, degree of coronary artery stenosis, number of coronary artery lesions, and Gensini score, and corresponding Gensini scores for each of the four major branches [left main stem (LM), left anterior descending (LAD) branch, left circumflex (LCX) branch, and right coronary artery (RCA) branch].

### 2.3 Instruments and Methods

#### 2.3.1 Immune Inflammatory Indicators 

All laboratory parameters were assessed in a standardized laboratory of the Department of Clinical Laboratory at Hefei Affiliated Hospital of Anhui Medical University. The following systemic immune inflammatory indices were calculated: systemic immune inflammation response index (SIIRI), systemic inflammatory response index (SIRI), systemic immune inflammation index (SII), neutrophil to lymphocyte ratio (NLR), platelet to lymphocyte ratio (PLR), monocyte to lymphocyte ratio (MLR), and neutrophil to high-density lipoprotein cholesterol ratio (NHR). The formulas for these indices are as follows: SIIRI = P × N × M / L, SII = P × N / L, SIRI = N × M / L, NLR = N / L, PLR = P / L, MLR = M / L, NHR = N / HDL-C, where P represents platelets, N represents neutrophils, M represents monocytes, L represents lymphocytes, and R represents the ratio of other specified blood components to lymphocytes.

#### 2.3.2 Ultrasound Examination 

Prior to surgery, all patients underwent ultrasound examination using the Aixplorer ultrasound diagnostic device (version V, SuperSonic Imagine, Aix-en-Provence, France), which is equipped with AP technology (version 12.3.1.849, SuperSonic Imagine, Aix-en-Provence, France) and an SL10-2 transducer (SSIP92085, SuperSonic Imagine, Aix-en-Provence, France) with a frequency range of 2 to 10 MHz. A conventional two-dimensional ultrasound examination was initially performed, with thorough exposure of the patient’s neck. The common carotid artery trunk, bifurcation, internal carotid artery, and external carotid artery on both sides were scanned sequentially in short and long axis views. IMT was measured at the lower 1.0 to 1.5 cm of the common carotid artery bifurcation. The transducer should be kept as parallel as possible to the arterial wall. The presence of plaques was assessed. According to the Chinese stroke vascular ultrasound examination guidelines, plaques were classified into homogeneous echogenicity (hypoechoic, isoechoic, hyperechoic) and heterogeneous echogenicity (with more than 20% inconsistent echogenicity) [9].

Subsequently, in the color doppler flow imaging (CDFI) mode, the AP technology was activated, and the colour power imaging (CPI) mode was selected. The size of the sampling frame was adjusted, observed for 30 s, determined whether there are point or line blood flow signals in the target plaque. The section with the richest blood flow signals was selected, and attention was given to exclude the artifacts of calcification. All the dynamic and static images were stored for further study. The AP technology parameters were modified as follows: Map 8, dynamic range 58 dB, low persistence, frame rate 14 Hz, and depth 3.6 cm.

**Fig. 1.**
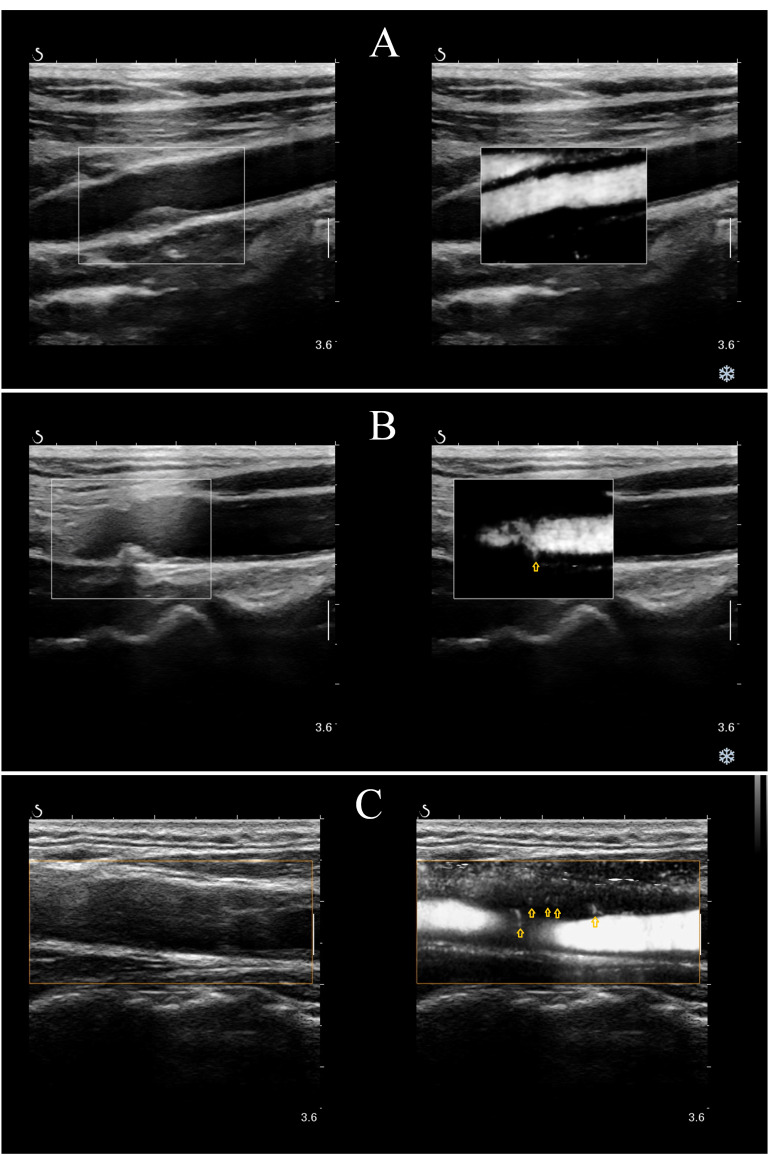
**Carotid IPN scoring method. Representative IPN images of carotid plaques**. Yellow arrows depict IPNs. (A) 0, no visible blood flow signal within the plaque. (B) 1, a small number of dotted or short line blood flow signals (less than 4) on one side of the plaque. (C) 2, short-lined, linear, diffuse dendritic blood flow signals in plaques (4 or more). IPN, intraplaque neovascularization.

A patient was considered to have IPN if any of his (or her) multiple plaques exhibit IPN. The IPN score is as follows: 0 points, no blood flow signals within the plaque; 1 point, a few punctate or short linear blood flow signals (less than 4) on one side of the plaque; 2 points, short linear, linear, or diffuse tree-like blood flow signals (4 or more) within the plaque (Fig. 1). A video of Fig. 1C. is available at the URL in the **Supplementary Materials**. Patients were then categorized into Group A (IPN = 0), Group B (IPN = 1), and Group C (IPN = 2) based on the IPN score. Ultrasound examinations were conducted by two senior physicians prior to the patients’ CAG and when their vital signs were stable. Both physicians were blinded to the patients’ clinical information. In cases where there was a discrepancy in the IPN scoring of the plaques, the final decision was based on a consensus agreement reached between the two experts.

#### 2.3.3 CAG

Researchers scored the coronary angiography findings without knowledge of the participants’ clinical characteristics. The Gensini scoring system was utilized to assess the degree of coronary artery stenosis, with the final score for each patient’s coronary artery disease being the sum of the products of the stenosis severity scores for each vessel segment and the corresponding vessel coefficients [10]. Patients were categorized into two groups based on the severity of coronary artery stenosis on angiography: mild and severe stenosis. The severe stenosis group was defined as having any major coronary branch with stenosis ≥70% or left main stem stenosis ≥50% [5]. The number of involved major coronary branches (LM, LAD, LCX, RCA) was also recorded. At the same time, the Gensini score was calculated for each major coronary artery.

### 2.4 Statistical Analyses 

Data collected were statistically analyzed using IBM SPSS Statistics 25.0 software (IBM, Armonk, NY, USA), and graphs were generated with GraphPad Prism 8.0.1 software (GraphPad Prism Software Inc., San Diego, CA, USA). All tests were two sided, and a *p* < 0.05 was considered to be statistically significant. Quantitative data were tested for normality using the Shapiro-Wilk test. Data conforming to a normal distribution were described using the mean ± standard deviation, and comparisons between two groups were made using the independent samples *t*-test. For comparisons among multiple groups, one-way analysis of variance was applied. Non-normally distributed data were described using the median (first quartile, third quartile), and comparisons between two groups were made using the Mann-Whitney U test. For multiple group comparisons, the Kruskal-Wallis H test was used, followed by pairwise comparisons with Bonferroni correction. Categorical and ordinal data were expressed as percentages, and differences were analyzed using the χ^2^ test, corrected χ^2^, or Fisher’s exact test. Correlations were assessed using Spearman’s rank correlation analysis. Immune inflammatory indicators and plaque’s two-dimensional characteristics that showed significant differences between groups with and without IPN were subjected to univariate logistic regression analysis. Variables with a *p* < 0.05 were included in a multivariate logistic regression analysis to determine independent predictors of IPN. The predictive value of factors influencing IPN was assessed by plotting the receiver operating characteristic (ROC) curve. Odds ratios (ORs) and 95% confidence intervals (CIs) were calculated.

## 3. Results 

### 3.1 Clinical Characteristics of Groups With and Without IPN

**Fig. 2.**
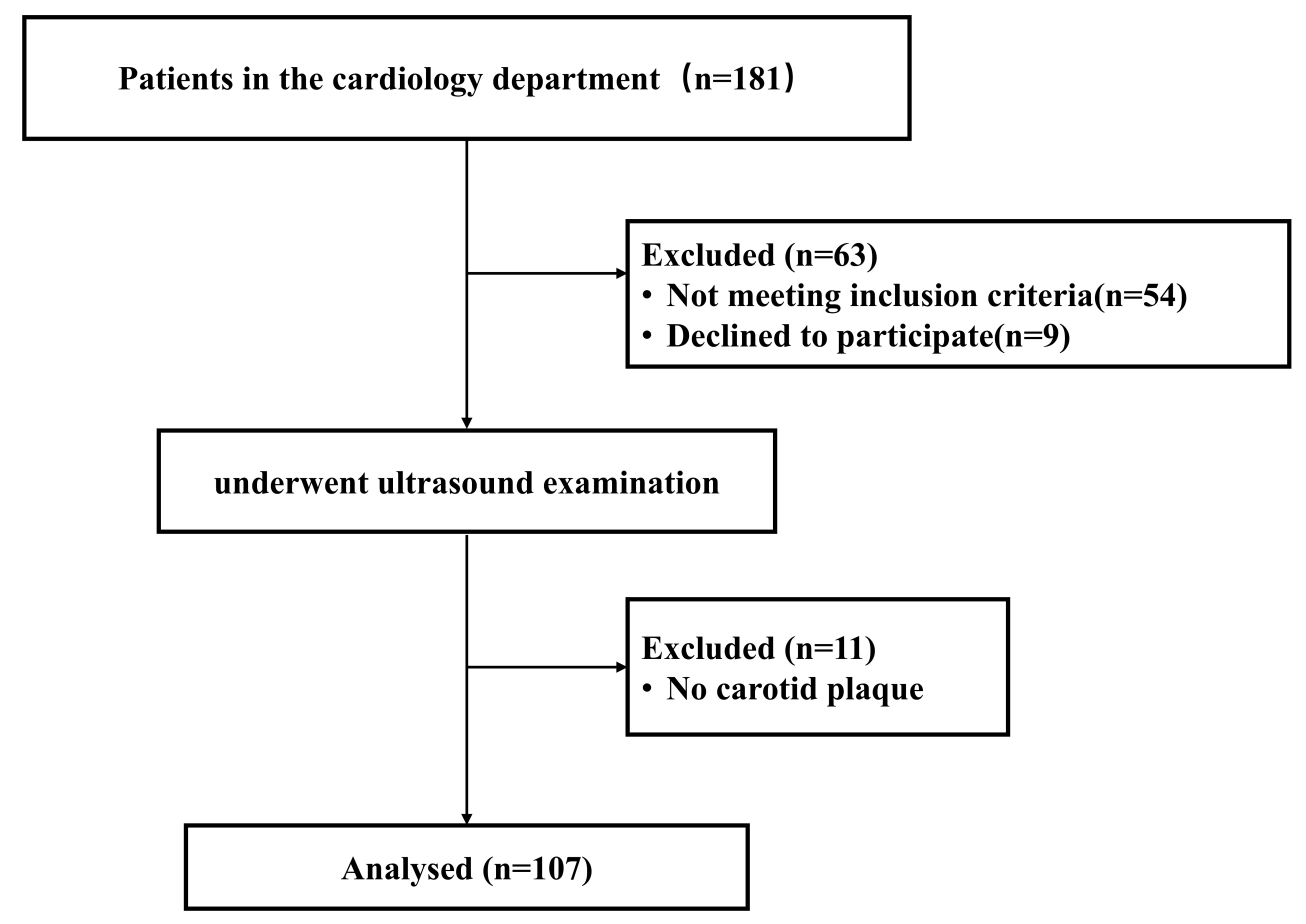
**Patient inclusion and exclusion procedures**.

Of the 181 participants screened, 54 did not meet the study’s inclusion and exclusion criteria, and 9 declined to participate (Fig. 2). Ultrasound examinations were conducted on 118 participants. 11 participants were excluded due to the absence of carotid plaque. Overall, the study included 107 participants. Among them, 54 had no evidence of IPN, while 53 had IPN.

The baseline characteristics of the study population are presented in Table 1. In the study population, 64 cases (59.8%) were males. Atrial fibrillation was present in 7.5%, stroke in 29.9%, smoking in 30.8%, alcohol consumption in 23.4%, hypertension in 79.4% hyperlipidemia in 63.6%, diabetes mellitus in 34.6%, and 54 patients have taken statins. Except for SIIRI, SIRI, SII, NHR, the degree of coronary artery stenosis, Gensini score, Gensini score for each coronary artery, the number of coronary artery diseased vessels, and plaque thickness; no statistically significant differences were observed between the two groups for other variables (*p* > 0.05). The IPN group had significantly higher levels of SIIRI, SIRI, SII, NHR, a greater degree of coronary artery stenosis, higher Gensini scores, and number of diseased vessels compared to the non-IPN group (*p* < 0.05). The plaque thickness was significantly greater in the IPN group than in the non-IPN group (*p* < 0.05). However, there were no statistically significant differences in carotid intima-media thickness and plaque length between the two groups (*p* > 0.05). In terms of plaque echogenicity, there were no statistically significant differences in distribution between the two groups (*p* > 0.05).

**Table 1.** **Comparison of clinical data among Patients with or without IPN**.

Variable			Patients without IPN	Patients with IPN	t/χ^2^*/*Z	*p* value
			(n = 54)	(n = 53)		
Age, years			68.26 ± 10.13	67.91 ± 9.43	0.187	0.852
Sex (Male)			34 (63.0%)	30 (56.6%)	0.450	0.502
BMI, kg/m^2^			25.21 ± 3.19	24.87 ± 2.97	0.573	0.568
Clinical history, n (%)						
	Atrial fibrillation		6 (11.1%)	2 (3.8%)	1.156	0.282
	Stroke		16 (29.6%)	16 (30.2%)	0.004	0.950
	Smoking		14 (25.9%)	19 (35.8%)	1.235	0.266
	Drinking		13 (24.1%)	12 (22.6%)	0.031	0.861
	Hypertension		41 (75.9%)	44 (83.0%)	0.824	0.364
	Hyperlipidemia		31 (57.4%)	37 (69.8%)	1.777	0.183
	Diabetes mellitus		19 (35.2%)	18 (34.0%)	0.018	0.894
Concomitant medication, n (%)						
	Statins		24 (44.4%)	30 (56.6%)	1.582	0.208
Laboratory assessment						
	TG, mmol/L		1.33 (1.02, 1.76)	1.38 (0.96, 1.86)	–0.081	0.935
	TC, mmol/L		4.38 (3.36, 5.00)	4.31 (3.31, 5.09)	–0.016	0.988
	HDL-C, mmol/L		1.16 (0.99, 1.36)	1.09 (0.90, 1.32)	–1.334	0.182
	LDL-C, mmol/L		2.76 (2.06, 3.21)	2.87 (2.06, 3.36)	–0.421	0.674
	Cr, umol/L		68.75 (59.18, 84.33)	66.20 (56.45, 80.50)	–0.723	0.470
	UA, umol/L		336.45 (254.75, 410.28)	348.20 (285.25, 397.05)	–0.617	0.537
	FBG, mmol/L		5.46 (4.89, 6.49)	5.91 (5.07, 7.35)	–1.259	0.208
	SIIRI, 10^18^/L^2^		159.50 (103.00, 250.25)	244.00 (168.50, 340.00)	–3.165	0.002
	SIRI, 10^9^/L		0.90 (0.62, 1.28)	1.08 (0.87, 1.53)	–2.493	0.013
	SII, 10^9^/L		463.50 (367.25, 655.25)	630.00 (431.50, 839.50)	–2.726	0.006
	NLR		2.57 (1.96, 3.14)	2.83 (2.24, 4.50)	–1.573	0.116
	PLR		121.50 (100.50, 145.25)	121.00 (108.00, 182.50)	–1.109	0.267
	MLR		0.23 (0.18, 0.28)	0.26 (0.19, 0.37)	–1.771	0.077
	NHR, 10^9^/mmol		3.37 (2.45, 4.76)	3.96 (3.35, 5.75)	–2.486	0.013
Coronary artery						
	Degree of coronary artery stenosis, n (%)				18.301	<0.001
		Mild	32 (59.3%)	10 (18.9%)		
		Severe	22 (40.7%)	43 (81.1%)		
	Gensini score		11 (5.00, 24.25)	35 (14.00, 50.50)	–3.987	<0.001
	Gensini score for LM		0 (0, 0)	0 (0, 0)	–2.186	0.029
	Gensini score for LAD		5 (2.5, 11.5)	12 (5, 28.5)	–2.704	0.007
	Gensini score for LCX		5 (0, 13)	9 (2, 32.8)	–2.017	0.044
	Gensini score for RCA		1 (0, 4)	4 (0, 12)	–2.878	0.004
	Number of coronary artery diseased vessels, n (%)				Fisher	0.003
		1	24 (44.4%)	15 (28.3%)		
		2	15 (27.8%)	7 (13.2%)		
		3	15 (27.8%)^a^	25 (47.2%)		
		4	0 (0.0%)^a^	6 (11.3%)		
Plaque						
	IMT, mm		0.89 ± 0.16	0.91 ± 0.19	–0.685	0.495
	Thickness, mm		2.65 (1.98, 3.33)	3.00 (2.50, 3.65)	–2.778	0.005
	Length, mm		9.30 (5.88, 14.10)	10.50 (8.45, 14.85)	–1.932	0.053
	Echo				3.623	0.305
	Uniform hypoecho		16 (29.6%)	11 (20.8%)		
	Homogeneous Isoechoic echo		9 (16.7%)	5 (9.4%)		
	Uniform hyperecho		6 (11.1%)	5 (9.4%)		
	Uneven echo		23 (42.6%)	32 (60.4%)		

^a^Compared to patients with IPN, *p* < 0.05. BMI, body mass index; Cr, creatinine; FBG, fasting blood glucose; HDL-C, high-density lipoprotein cholesterol; IMT, intima-media thickness; IPN, intraplaque neovascularization; LDL-C, low-density lipoprotein cholesterol; LAD, left anterior descending; LCX, left circumflex; LM, left main stem; MLR, monocyte to lymphocyte ratio; NHR, neutrophil to HDL-C ratio; NLR, neutrophil to lymphocyte ratio; PLR, platelet to lymphocyte ratio; RCA, right coronary artery; SII, systemic immune inflammation index; SIIRI, systemic immune inflammation response index; SIRI, systemic inflammatory response index; TC, total cholesterol; TG, triglyceride; UA, uric acid.

### 3.2 Characteristics of Populations With Different IPN Scores

There were statistically significant differences in the overall distribution of SIIRI, SIRI, SII, NHR, the degree of coronary artery stenosis, Gensini score, the number of coronary artery diseased vessels, plaque thickness, and plaque length among the three groups (*p* < 0.05). The differences in the other indicators were not statistically significant (*p* > 0.05) (Table 2).

**Table 2.** **Comparison of clinical data among different IPN scores**.

Variable			Group A	Group B	Group C	*F*/χ^2^/*H*	*p* value
			(n = 54)	(n = 38)	(n = 15)		
Age, years			68.26 ± 10.13	67.84 ± 9.76	68.07 ± 8.87	0.020	0.980
Gender (Male)			34 (63.0%)	20 (52.6%)	10 (66.7%)	1.331	0.514
BMI, kg/m^2^			25.21 ± 3.19	24.72 ± 2.85	25.25 ± 3.32	0.319	0.728
Clinical history, n (%)							
	Atrial fibrillation		6 (11.1%)	0 (0%)	2 (13.3%)	Fisher	0.054
	Stroke		16 (29.6%)	13 (34.2%)	3 (20.0%)	Fisher	0.621
	Smoking		14 (25.9%)	12 (31.6%)	7 (46.7%)	2.383	0.304
	Drinking		13 (24.1%)	6 (15.8%)	6 (40.0%)	3.551	0.169
	Hypertension		41 (75.9%)	33 (86.8%)	11 (73.3%)	2.025	0.363
	Hyperlipidemia		31 (57.4%)	26 (68.4%)	11 (73.3%)	1.889	0.389
	Diabetes mellitus		19 (35.2%)	12 (31.6%)	6 (40.0%)	0.355	0.837
Concomitant medication, n (%)							
	Statins		24 (44.4%)	19 (50.0%)	11 (73.3%)	3.924	0.141
Laboratory assessment							
	TG, mmol/L		1.33 (1.02, 1.76)	1.48 (1.11, 1.93)	1.05 (0.82, 1.53)	1.908	0.385
	TC, mmol/L		4.38 (3.36, 5.00)	4.31 (3.32, 5.51)	4.31 (3.05, 4.91)	0.206	0.902
	HDL-C, mmol/L		1.16 (0.99, 1.36)	1.06 (0.88, 1.36)	1.13 (0.91, 1.23)	1.796	0.407
	LDL-C, mmol/L		2.76 (2.06, 3.21)	2.87 (2.16, 3.34)	2.53 (1.92, 3.50)	0.328	0.849
	Cr, umol/L		68.75 (59.18, 84.33)	66.10 (56.75, 80.35)	69.30 (55.80, 84.80)	0.560	0.756
	UA, umol/L		338.46 ± 106.25	361.43 ± 100.76	341.49 ± 85.65	0.594	0.554
	FBG, mmol/L		5.46 (4.89, 6.49)	5.95 (5.11, 7.26)	5.84 (4.92, 7.77)	1.643	0.440
	SIIRI, 10^18^/L^2^		159.50 (103.00, 250.25)^a,b^	245.00 (156.00, 339.50)	236.00 (205.00, 348.00)	10.551	0.005
	SIRI, 10^9^/L		0.90 (0.62, 1.28)	1.05 (0.82, 3.14)	1.33 (0.97, 2.41)	7.044	0.030
	SII, 10^9^/L		463.50 (367.25, 655.25)	619.00 (398.50, 826.50)	665.00 (474.00, 847.00)	7.699	0.021
	NLR		2.57 (1.96, 3.14)	2.56 (2.24, 4.63)	3.15 (2.22, 3.73)	3.094	0.213
	PLR		121.50 (100.50, 145.25)	121.00 (103.75, 175.25)	124.00 (114.00, 198.00)	1.254	0.534
	MLR		0.23 (0.18, 0.28)	0.26 (0.19, 0.39)	0.26 (0.22, 0.32)	3.325	0.190
	NHR, 10^9^/mmol		3.37 (2.45, 4.76)	3.85 (3.13, 5.71)	4.81 (3.44, 6.27)	6.762	0.034
Coronary artery							
	Degree of coronary artery stenosis, n (%)					21.424	<0.001
		Mild	32 (59.3%)^a,b^	10 (26.3%)	0 (0.0%)^c^		
		Severe	22 (40.7%)^a,b^	28 (73.7%)	15 (100.0%)^c^		
	Gensini score		11 (5, 24.25)^a,b^	21 (10, 47.5)^a,c^	48 (29, 70)^b,c^	22.866	<0.001
	Gensini score for LM		0 (0, 0)	0 (0, 0)	0 (0, 0)	4.781	0.092
	Gensini score for LAD		5 (2.5, 11.5)^a^	11 (4.75, 20.5)	18 (8, 40)^c^	8.263	0.016
	Gensini score for LCX		0 (0, 5)^a^	0 (0, 6)	5 (0, 20)^c^	7.895	0.019
	Gensini score for RCA		1 (0, 4)^a^	2 (0, 9)^a^	10 (4, 20)^c^	15.572	<0.001
	Number of coronary artery diseased vessels, n (%)					Fisher	0.004
		1	24 (44.4%)	13 (34.2%)	2 (13.3%)		
		2	15 (27.8%)	6 (15.8%)	1 (6.7%)		
		3	15 (27.8%)^a^	15 (39.5%)	10 (66.7%)		
		4	0 (0.0%)^a,b^	4 (10.5%)	2 (13.3%)		
Plaque							
	IMT, mm		0.89 ± 0.16	0.88 ± 0.17	0.97 ± 0.23	1.559	0.215
	Thickness, mm		2.65 (1.98, 3.33)	3.05 (2.50, 3.60)	3.00 (2.50, 4.20)	7.987	0.018
	Length, mm		9.30 (5.88, 14.10)^a^	9.75 (7.95, 13.90)	12.00 (9.60, 15.90)	6.227	0.044
	Echo					Fisher	0.348
	Uniform hypoecho		16 (29.6%)	10 (26.3%)	1 (6.7%)		
	Homogeneous isoechoic echo		9 (16.7%)	4 (10.5%)	1 (6.7%)		
	Uniform hyperecho		6 (11.1%)	4 (10.5%)	1 (6.7%)		
	Uneven echo		23 (42.6%)	20 (52.6%)	12 (80.0%)		

^a^Compared to Group C, *p* < 0.05. ^b^Compared to Group B, *p* < 0.05. ^c^Compared to Group A, *p* < 0.05. BMI, body mass index; Cr, creatinine; FBG, fasting blood glucose; HDL-C, high-density lipoprotein cholesterol; IMT, intima-media thickness; IPN, intraplaque neovascularization; LDL-C, low-density lipoprotein cholesterol; LAD, left anterior descending; LCX, left circumflex; LM, left main stem; MLR, monocyte to lymphocyte ratio; NHR, neutrophil to HDL-C ratio; NLR, neutrophil to lymphocyte ratio; PLR, platelet to lymphocyte ratio; RCA, right coronary artery; SII, systemic immune inflammation index; SIIRI, systemic immune inflammation response index; SIRI, systemic inflammatory response index; TC, total cholesterol; TG, triglyceride; UA, uric acid.

Gensini score were statistically different between the three groups (*p* < 0.05). Compared to Groups B and C, Group A exhibited statistically significant differences in SIIRI, the degree of coronary artery stenosis, Gensini score, and the involvement of all four coronary arteries (*p* < 0.05). Furthermore, when comparing Group A to Group C, there were statistically significant differences in the involvement of the three coronary arteries and plaque length (*p* < 0.05). Post-hoc pairwise comparison analysis with Bonferroni correction for significance revealed no statistically significant differences in SIRI, SII, NHR, and plaque thickness among the three groups (adjusted *p* > 0.017).

### 3.3 Correlation between IPN Score with Gensini Score and Immune Inflammatory Biomarkers

**Fig. 3.**
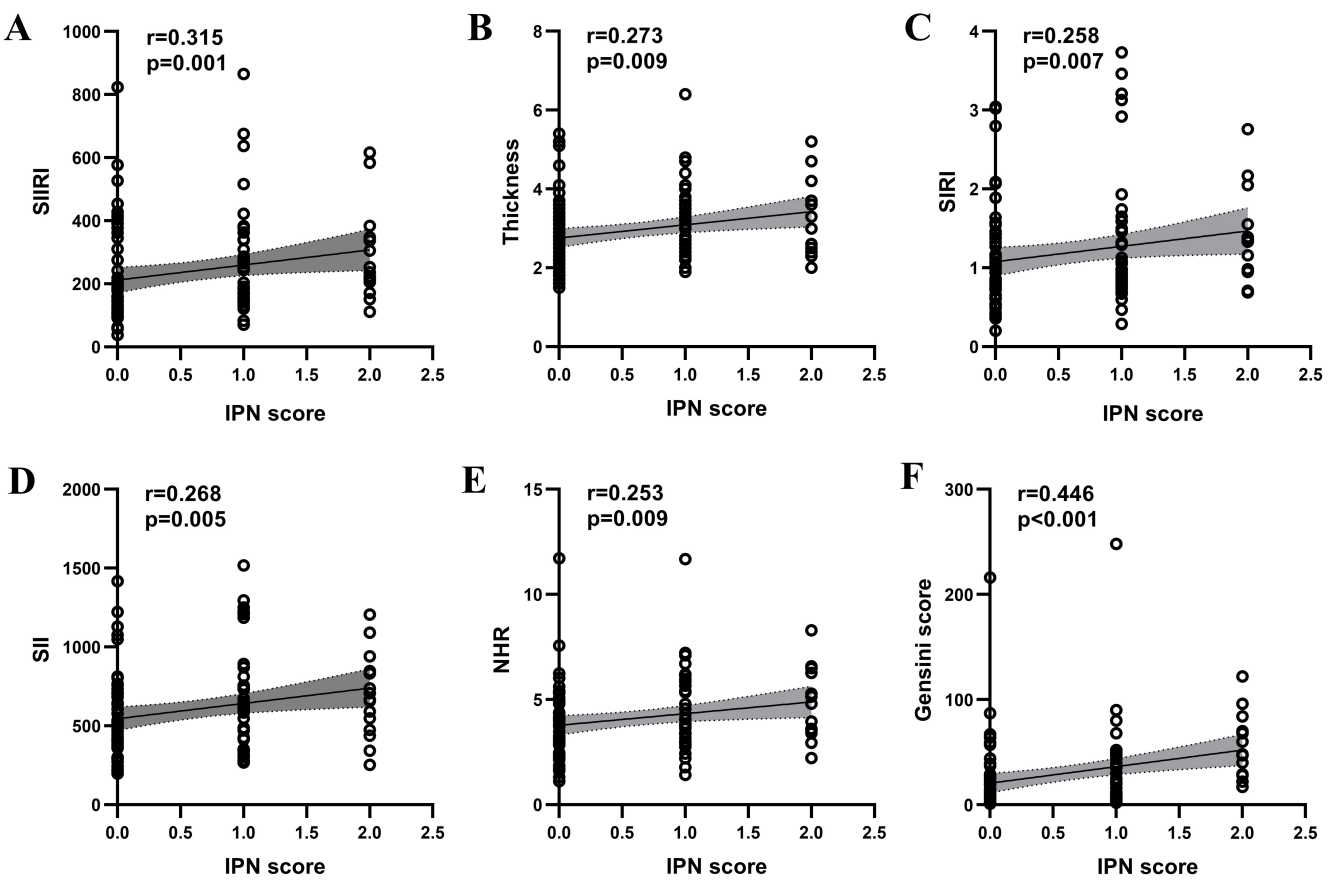
**Spearman correlation analysis of IPN scores with SIIRI (A), thickness (B), SIRI (C), SII (D), NHR (E) and Gensini Score (F) in patients with atheromatous carotid artery plaque**. IPN, intraplaque neovascularization; NHR, neutrophil to HDL-C ratio; r, correlation coefficient; SII, systemic immune inflammation index; SIIRI, systemic immune inflammation response index; SIRI, systemic inflammatory response index.

**Fig. 4.**
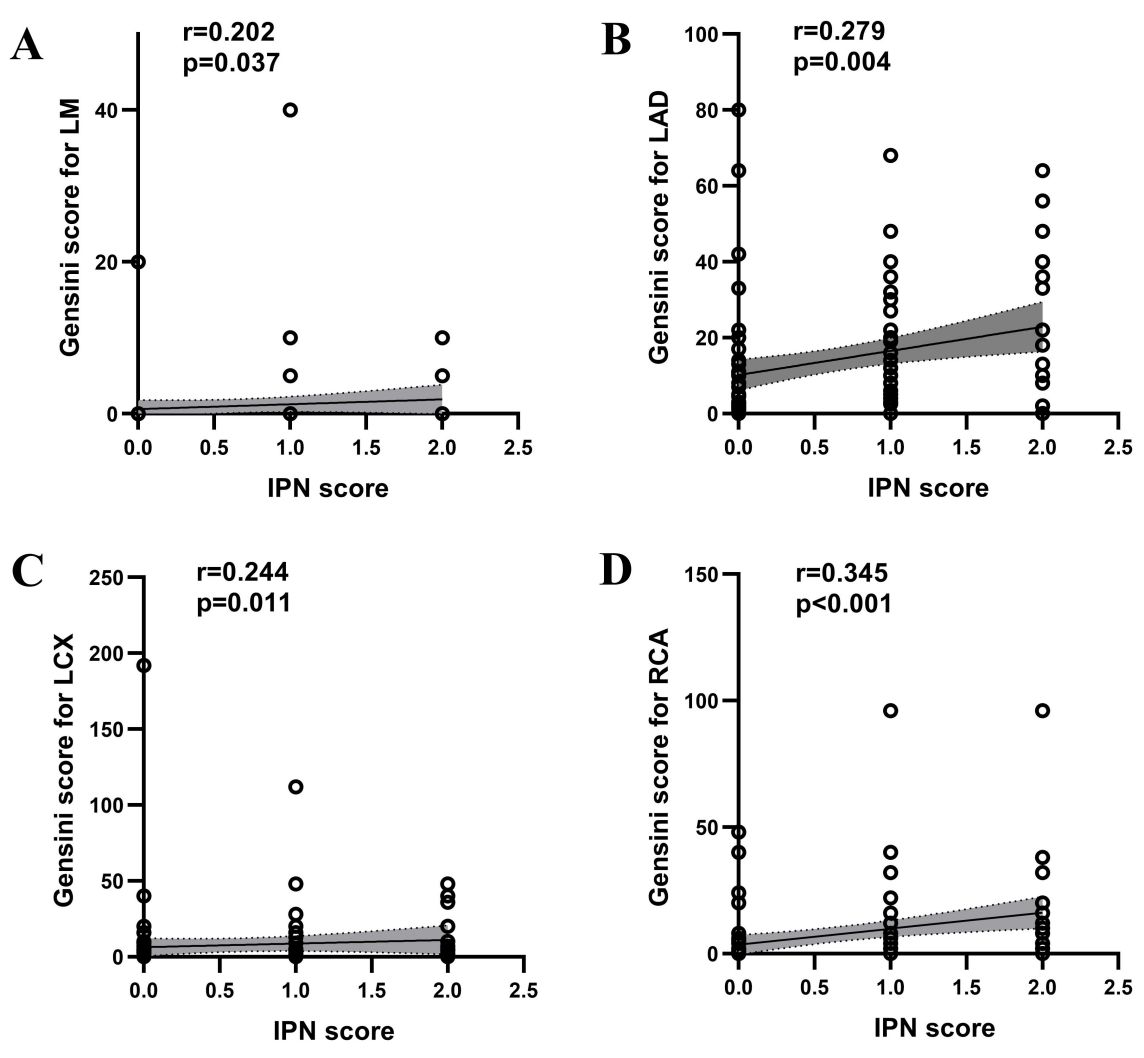
**Spearman correlation analysis of IPN with Gensini Score for LM (A), Gensini Score for LAD (B), Gensini Score for LCX (C) and Gensini Score for RCA (D) in patients with atheromatous carotid artery plaque**. IPN, intraplaque neovascularization; LM, left main stem; LAD, left anterior descending; LCX, left circumflex; r, correlation coefficient; RCA, right coronary artery.

The IPN score demonstrated positive correlations with the SIIRI (r = 0.315, *p* = 0.001), plaque thickness (r = 0.273, *p* = 0.005), SIRI (r = 0.258, *p* = 0.007), SII (r = 0.268, *p* = 0.005), and NHR (r = 0.253, *p* = 0.009) (Fig. 3). Additionally, a significant positive correlation was observed between the IPN score and the Gensini score (r = 0.446, *p* < 0.001) (Fig. 3). The results also indicated significant correlations between the Gensini Score for LAD, LCX, RCA with the IPN score (LAD: r = 0.279, *p* = 0.004; LCX: r = 0.244, *p* = 0.011; RCA: r = 0.345, *p* < 0.001). The LM showed a weaker correlation with the IPN score (LM: r = 0.202, *p* = 0.037) (Fig. 4).

### 3.4 Univariate and Multivariate Logistic Regression Analysis in Individuals With IPN

In the univariate logistic regression analysis, SIIRI, SIRI, SII, NHR, and plaque thickness were found to be statistically significant (*p* < 0.05) (Table 3). All factors with significance in the univariate analysis were included in the multivariate logistic regression analysis. Since the OR represents the change in the odds of the outcome for a one-unit increase in the predictor, a one-unit change in SIIRI or SII might have a minimal effect on the outcome. To mitigate this, we divided the independent variables (SIIRI and SII) by a factor of 10 and then performed logistic regression analysis again. This adjustment addresses the issue of variability and ensures that the ORs reflect clinically relevant changes in the predictors.

**Table 3.** **Univariate logistic regression analyses of independent predictors of the presense of IPN**.

Predictor	β	SE	Wald χ^2^	OR (95% CI )	*p *value
SIIRI	0.030	0.014	4.801	1.031 (1.003∼1.059)	0.028
SIRI	0.639	0.305	4.404	1.895 (1.043∼3.442)	0.036
SII	0.019	0.007	6.506	1.019 (1.004∼1.033)	0.011
NHR	0.265	0.122	4.715	1.304 (1.026∼1.657)	0.030
Thickness	0.544	0.223	5.949	1.723 (1.113∼2.667)	0.015

CI, confidence interval; IPN, intraplaque neovascularization; NHR, neutrophil to HDL-C ratio; OR, odds ratio; SE, standard error; SII, systemic immune inflammation index; SIIRI, systemic immune inflammation response index; SIRI, systemic inflammatory response index.

The results indicated that SII (per 10-unit increase) (OR = 1.031, 95% CI: 1.002~1.059) and plaque thickness (OR = 1.897, 95% CI: 1.192~3.018) were independent risk factors for the presence of IPN (Table 4).

**Table 4.** **Multivariate logistic regression analyses of independent predictors of the presense of IPN**.

Predictor	β	SE	Wald χ²	OR (95% CI )	*p *value
SIIRI	–0.032	0.041	0.591	0.969 (0.894∼1.050)	0.442
SIRI	–0.047	0.786	0.004	0.955 (0.205∼4.452)	0.953
SII	0.030	0.014	4.559	1.031 (1.002∼1.059)	0.033
NHR	0.281	0.155	3.283	1.325 (0.977∼1.796)	0.070
Thickness	0.640	0.237	7.305	1.897 (1.192∼3.018)	0.007

CI, confidence interval; IPN, intraplaque neovascularization; NHR, neutrophil to HDL-C ratio; OR, odds ratio; SE, standard error; SII, systemic immune inflammation index; SIIRI, systemic immune inflammation response index; SIRI, systemic inflammatory response index.

### 3.5 Value of SII and Plaque Thickness as a Risk Stratification Tool for the Vulnerable Plaque

**Fig. 5.**
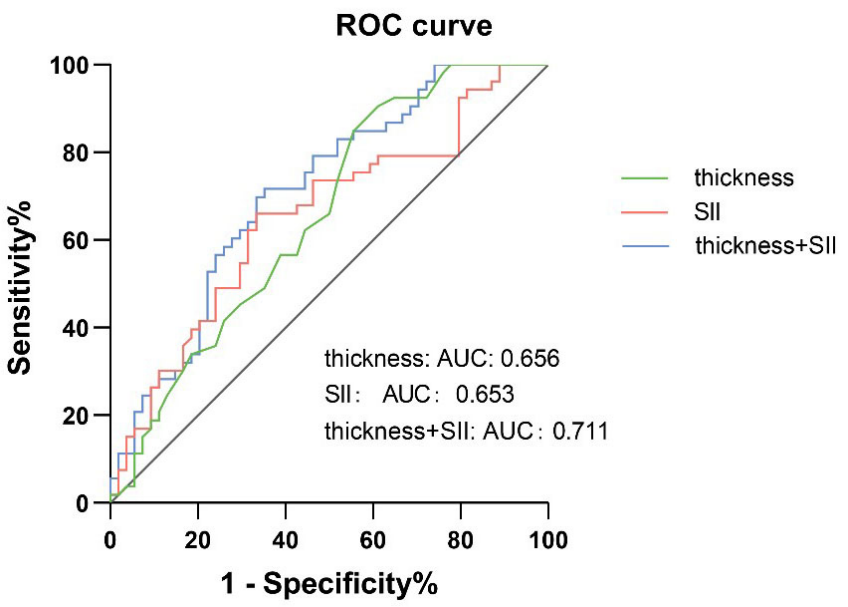
**ROC curve describing the diagnostic performance of SII and thickness to identify the presense of IPN**. AUC, area under the curve; IPN, intraplaque neovascularization; ROC, receiver operating characteristic; SII, systemic immune inflammation index.

ROC curves were constructed based on the patients’ SII levels and plaque thickness. The analysis indicated that the optimal cutoff points for SII and plaque thickness were 541 × 10^9^/L and 2.25 mm, respectively, yielding the highest Youden’s index. The respective area under curves (AUC) were 0.653 (95% CI: 0.548~0.757, *p* = 0.006) and 0.656 (95% CI: 0.552~0.759, *p* = 0.006). The sensitivities were 66.04% and 90.57%, while the specificities were 66.07% and 38.89%. When both parameters were used in conjunction for diagnosis, the AUC was 0.711 (95% CI: 0.614~0.808, *p* < 0.001), with a sensitivity of 71.70% and a specificity of 64.82% (Fig. 5).

## 4. Discussion

This prospective investigation has identified a correlation between SII levels in peripheral blood and the thickness of carotid plaques with the vulnerability of these plaques in patients with varying degrees of coronary artery stenosis, as confirmed by CAG. Both SII levels and carotid plaque thickness were determined to be independent risk factors for carotid plaque vulnerability. In addition, the combined application of these two parameters improved the predictive accuracy of carotid vulnerable plaques in patients with coronary artery disease. Additional correlation analyses have demonstrated a positive association between the IPN score and the Gensini score. This suggests that an increase in plaque vulnerability is paralleled by a greater degree of coronary artery narrowing.

Intraplaque hemorrhage is often associated with the leakage of microvessels within atherosclerotic plaques [11]. AP technology, an innovative ultrasound method, measures microvascular blood flow by utilizing the acoustic radiation force of ultrasound waves to induce minute vibrations within tissues. These vibrations are detected by a synchronized ultrasound transducer, allowing for the quantification of blood flow direction and velocity. By analyzing the characteristics of these vibrations, AP technology can ascertain blood flow direction which provides clinicians with a novel tool for assessing plaque stability, blood supply, and cardiovascular risk [7]. Several studies have demonstrated AP’s ability to detect microvascular signals within plaques, clearly delineating the direction of blood flow within these microvessels, which are significant predictors of intraplaque hemorrhage [6, 7, 12].

Conventional ultrasound is a widely utilized technique for evaluating carotid IMT, plaque echo characteristics, length, and thickness. In our study, significant disparities in plaque thickness were observed between patients with and without IPN and across various IPN scores, aligning with the research of Zhang *et al*. [7] and Chen *et al*. [6]. Contrary to Kim *et al*. [11], who reported no significant difference in plaque thickness between IPN and non-IPN groups, we noted that plaques in the IPN group tended to be thicker. In terms of carotid IMT, although patients with IPN exhibited greater IMT values compared to those without IPN, the difference was not statistically significant, corroborating the findings of Zhang *et al*. [7] and Kim *et al*. [11]. This lack of statistical significance might be attributed to the limited sample size and potential bias from the examiner’s subjective assessments. Echogenicity of the plaques did not significantly differ between patients with and without IPN or among different IPN scores, a finding that diggers from Zhang *et al*. [7]. Given the propensity for plaques to calcify over time, such plaques in this study were predominantly categorized as having heterogeneous echogenicity. Furthermore, no significant differences in plaque length were detected, suggesting that plaque vulnerability may not be associated with increased length but is more likely related to thickness.

In this study, significant disparities were observed in the number of vessels affected by coronary artery disease and the severity of coronary artery stenosis among patients with and without IPN, as well as among patients with varying IPN scores. This finding reveals the complexity of coronary artery lesions and their association with IPN. Additionally, the Spearman correlation analysis between the IPN score and the Gensini score demonstrated a correlation, reinforcing the notion that AS is a systemic and widespread pathological process. This is in line with the findings of Mantella *et al*. [5], which underscores the significance of evaluating the overall vascular health in patients with AS. Consequently, the assessment of IPN could potentially identify patients at an elevated risk for coronary artery events and may help to develop more precise therapeutic approaches.

AS, a systemic chronic inflammatory condition affecting the arterial wall, involves complex interactions among endothelial cells, immune cells, and vascular smooth muscle cells [13, 14]. Throughout the progression of AS, cholesterol and lipids debris in the bloodstream accumulate at sites of endothelial injury, potentially leading to cholesterol oxidation and the initiation of inflammatory responses [15]. Studies have established that inflammation is a consistent consistent feature throughout the pathogenesis of AS [16]. Endothelial damage facilitates the deposition of plasma constituents in the intima, which in turn triggers platelet adhesion, aggregation, and the secretion of multiple bioactive substances. These substances recruit monocytes to the endothelium and encourage their transmigration into the subintimal space [17]. Inflammatory mediators released by neutrophils contribute to endothelial dysfunction and vascular wall deterioration, while also promoting the recruitment and activation of monocytes, macrophages, and dendritic cells, thereby intensifying the inflammatory response and AS [18, 19]. The infiltration of neutrophils, monocytes, and lymphocytes in AS initiates a cytokine-driven inflammatory cascade that can destabilize plaques and potentially precipitate cardiovascular events. Lymphocytes play an essential role in modulating immune responses and curbing excessive inflammation, thereby maintaining a critical balance [20].

The immune inflammatory mechanisms underlying AS encompass a spectrum of indicators, accessible through routine blood testing, which have been instrumental in the evaluation of cardiovascular and cerebrovascular diseases [21, 22, 23, 24]. In this study, we selected a panel of indicators including SIIRI, SIRI, SII, NLR, PLR, MLR, and NHR for analysis. Significant differences were observed in SIIRI, SIRI, SII, and NHR among patients with and without IPN, as well as across various IPN scores. However, post hoc pairwise comparisons adjusted for multiple testing using the Bonferroni method indicated that the differences in SIRI, SII, NHR, and plaque thickness among the groups were not statistically significant (adjusted *p* > 0.017). While the sample size was adequate to detect at least one group differing from the others, pinpointing the specific inter-group differences was challenging, primarily due to the inherent risk of type I errors associated with multiple comparisons.

Our research indicates that higher IPN scores are associated with elevated levels of immune inflammatory indicators. Specifically, there is a positive correlation between IPN scores and indicators such as SIIRI, SIRI, SII, NHR, and plaque thickness. Additionally, a positive correlation was observed between IPN scores and the Gensini score. In univariate logistic regression analysis, these indicators, along with plaque thickness, correlated with the vulnerability of plaques identified by AP technology. However, in multivariate logistic regression analysis, only SII (per 10-unit increase) (OR = 1.031, 95% CI: 1.002~1.059) and plaque thickness (OR = 1.897, 95% CI: 1.192~3.018) remained significantly associated with plaque vulnerability, suggesting they are independent risk factors for IPN. Consistent with our findings, Kim *et al*. [11] reported a significant association between matrix metalloproteinase-9 levels in plasma (OR = 1.014, 95% CI: 1.002~1.027) and IPN in patients with stable coronary heart disease. The potential mechanism underlying our results is that IPN leakage may facilitate the entry of inflammatory cells into the plaque. The increased fragility of the arterial wall affected by IPN, coupled with the loosening of endothelial cell junctions, could enhance the infiltration of inflammatory cells into the plaque, thereby promoting intraplaque hemorrhage and rupture.

SII, a novel indicator derived from the NLR and incorporating platelet and monocyte counts, reflects the body’s inflammatory status and immune balance. Research indicates that SII correlates significantly with the severity of CAD in patients with stable angina [25]. Elevated SII levels are also observed in patients with Acute Coronary Syndrome, where it serves as a predictor of CAD severity [22]. Furthermore, SII has been identified as a prognostic marker for Major Adverse Cardiovascular Events in individuals with myocardial infarction and non-obstructive coronary arteries [26]. In patients with an acute internal carotid artery stroke, higher SII levels have been associated with a greater prevalence of vulnerable carotid plaques [24]. SII has also been established as an independent risk factor for ulcerative plaques in ischemic stroke patients [27]. Elevated SII levels are further linked to an increased risk of stroke and all-cause mortality [28]. Collectively, these findings suggest that increased SII levels may signify the vulnerability and risk of rupture in atherosclerotic plaques, as well as its association with coronary artery stenosis. SII has been shown to be more clinically valuable than NLR, PLR, and MLR alone in predicting the severity of coronary artery disease, atherosclerosis, and other cardiovascular diseases [25]. In multivariate analysis, the SII, being a comprehensive index, can better adjust for the effects of other variables and maintain statistical significance. Because the SII consists of the ratio of platelet count, leukocyte count, and neutrophil count, it is capable of reflecting both aspects of the immune and inflammatory response, and is therefore more representative than a single inflammatory index (e.g., NLR, PLR, MLR). Elevated neutrophils and platelets suggest activation of inflammation, promoting plaque instability and neovascularization. Decreased lymphocytes suggest impaired immune function, further exacerbating disease progression. Therefore, SII is more advantageous in capturing the pathological mechanisms of neovascularization and coronary artery stenosis within atherosclerotic plaques. NLR, PLR, and MLR are all individually calculated ratios that may be highly correlated with each other (e.g., neutrophils and platelets are often elevated at the same time in response to inflammation), which may lead to multicollinearity, causing them to compete in multivariate models and affecting statistical significance. SII, as a composite metric, is able to avoid this problem.

IPN and the SII are both indicators of plaque vulnerability. While plaque thickness exhibits greater sensitivity than SII in identifying vulnerable plaques, its specificity is comparatively lower. This may be attributed to the study’s focus on a high-risk population and the natural progression of AS with increasing age. Although SIIRI, SIRI, and NHR did not achieve statistical significance in the multivariate logistic regression analysis, this study provides evidence that these immune inflammatory markers are valuable for detecting the presence of vulnerable plaques in the carotid artery.

## 5. Limitations

This study has several limitations. First, as a single-center investigation with a modest sample size, the statistical power is limited. Consequently, future research with larger cohorts and multi-center designs is warranted to bolster the external validity and reliability of the findings. Second, despite the presence of high-risk plaques in some participants, no adverse cardiovascular events were recorded during the study’s duration. This limitation hinders the assessment of the predictive utility of immune inflammatory markers and IPN in forecasting cardiovascular events. Long-term follow-up studies are necessary to ascertain the predictive value of these markers by monitoring the progression and evolution of carotid plaques. Third, in some patients with acute coronary syndromes treated with emergency surgery, routine ultrasound of carotid plaque and IPN were not available.

## 6. Conclusion

In conclusion, this study underscores the correlation between elevated SII levels and increased carotid plaque thickness with IPN, both of which are associated with the vulnerability of carotid plaques in patients with coronary artery stenosis and may exacerbate the severity of coronary stenosis. In addition, the association of increased SII levels and plaque thickness with IPN provides value to community hospitals in recognizing carotid plaque vulnerability through easily available immune inflammatory markers and assists in early detection and management of coronary stenosis in patients to improve their prognosis.

## Data Availability

The datasets used and/or analyzed in this study are available upon reasonable request from the corresponding author, Xiao Yang. These data are not publicly available because they contain information that may compromise patient privacy.

## Abbreviations

AP, Angio Planewave UltraSensitive imaging; AS, atherosclerosis; AUC, area under the curve; BMI, body mass index; CAD, coronary artery disease; CAG, coronary angiography; CDFI, color doppler flow imaging; CEUS, contrast-enhanced ultrasound; CI, confidence interval; CPI, colour power imaging; HDL-C, high-density lipoprotein cholesterol; IPN, intraplaque neovascularization; LAD, left anterior descending; LCX, left circumflex; LDL-C, low-density lipoprotein cholesterol; LM, left main stem; MLR, monocyte to lymphocyte ratio; NHR, neutrophil to HDL-C ratio; NLR, neutrophil to lymphocyte ratio; OR, odds ratio; PLR, platelet to lymphocyte ratio; r, correlation coefficient; RCA, right coronary artery; ROC, receiver operating characteristic; SE, standard error; SII, systemic immune inflammation index; SIIRI, systemic immune inflammation response index; SIRI, systemic inflammatory response index; TC, total cholesterol; TG, triglyceride.

## Supplementary Material

Supplementary material associated with this article can be found, in the online version, at https://doi.org/10.31083/RCM28171.
